# Closing Editorial: Special Issue on “Advances in Vaccine Adjuvants”

**DOI:** 10.3390/vaccines14090787

**Published:** 2026-09-09

**Authors:** Surabhi Gautam, Sanjeev Kumar, Devyani Joshi

**Affiliations:** 1 Department of Anatomy, Faculty of Medicine and Health Sciences, SGT University, Gurugram 122505, Haryana, India; drsurabhi_fmhs@sgtuniversity.org; 2 Department of Biochemistry, All India Institute of Medical Sciences, New Delhi 110029, Delhi, India; 3 Department of Pharmaceutical Sciences, School of Pharmacy, Massachusetts College of Pharmacy and Health Sciences, Boston, MA 02115, USA

As Guest Editors for the Special Issue “Advances in Vaccine Adjuvants,” we are pleased to offer this closing editorial. This Special Issue brings together eight contributions offering a timely overview of vaccine adjuvant research, spanning rational antigen–adjuvant co-design, comparative and mechanistic evaluation of adjuvant chemistries, novel adjuvanted delivery platforms, and lifespan-informed adjuvant strategy.

The capacity of adjuvants to shape durable, balanced humoral and cellular immunity has been consistently confirmed; however, the articles also highlight how antigen selection, carrier chemistry, and formulation stability jointly determine whether this benefit is realized in vivo. At the same time, work on microneedle- and intranasal-based delivery platforms reminds us that route of administration remains a critical and still underexploited lever for improving both immunogenicity and accessibility. Studies addressing age-specific adjuvant strategies, together with mechanistic investigations of adjuvant–antigen interactions, reflect the expanding role of adjuvant science beyond simple immune potentiation, toward precision-tailored, population-specific formulation design.

We would like to express our sincere gratitude to the *Vaccines* editorial team and all of the authors who contributed to this Special Issue for their invaluable assistance.

Shi et al. [1] combined the immunodominant toxoid antigen rLukS-PV with the subdominant adhesin antigen rClfA on a PLGA-PEG nanoparticle carrier to overcome the durability failures that have limited prior *Staphylococcus aureus* vaccine candidates. The combined nanovaccine elicited synergistic Th1/Th17-skewed immunity and sustained complete protection through Day 180, whereas the same antigens formulated with a conventional alum adjuvant failed to confer durable protection, underscoring the necessity of antigen–adjuvant co-design.

Zdrehus et al. [2] characterized carcinoembryonic antigen (CEA)-functionalized gold nanoparticles as a candidate nanovaccine platform, showing good cytocompatibility in murine macrophages and efficient cellular uptake with lysosomal trafficking consistent with productive antigen processing. These in vitro findings lay important groundwork for gold nanoparticles as tunable antigen carriers warranting further in vivo evaluation.

Ghattas et al. [3] directly compared chitosan, empty lipid nanoparticles, and aluminum hydroxide as adjuvants for a recombinant SARS-CoV-2 spike vaccine, finding that chitosan induced a more balanced Th1/Th2 response than alum, comparable to that seen with lipid nanoparticles, while driving a distinct cytokine/chemokine profile and a markedly greater recruitment of neutrophils. These results position chitosan as a versatile adjuvant candidate capable of rivaling lipid nanoparticle systems.

Kanjo et al. [4] investigated the biophysical interaction between a Class B CpG adjuvant and several proteins, including the SARS-CoV-2 receptor-binding domain, showing that CpG reduces thermal and proteolytic stability and promotes aggregation, particularly with prolonged incubation. This work highlights the need for systematic formulation stability studies alongside immunogenicity testing during adjuvant selection.

D’Onofrio et al. [5] reported a phase 1b trial of a novel carbohydrate fatty-acid monosulphate ester (CMS)-based squalane-in-water adjuvant combined with a quadrivalent influenza vaccine, demonstrating an acceptable safety profile and, notably, significantly higher hemagglutination inhibition titers in older adults relative to younger adults, which was an age-dependent immunogenicity benefit not observed with the unadjuvanted vaccine.

Arte et al. [6] developed a heterologous, alum-adjuvanted, PLGA-microencapsulated COVID-19 vaccine delivered via dissolving microneedles, using inactivated Delta virus as the priming antigen and Omicron virus as the booster, and observed cross-reactive antigen-specific antibody responses. These findings support microneedle-based cutaneous delivery as a practical route for variant-adapted, adjuvanted particulate vaccines.

Yao et al. [7] contributed a systematic review, grounded in an analysis of 127 peer-reviewed articles, of the mucosal barriers and adjuvant mechanisms relevant to intranasal vaccination, spanning pattern recognition receptor agonists, cytokine and cell-targeted adjuvants, and particulate delivery systems. The review identifies insufficient mucosal targeting and inconsistent global safety evaluation standards as key translational bottlenecks.

Singh et al. [8] reviewed licensed and late-stage viral vaccine adjuvants within an explicit age-stratified framework spanning neonates, infants, pregnancy, and older adults, underscoring that immune immaturity and immunosenescence each demand distinct adjuvant selection logic and identifying a persistent shortage of comparative, long-term safety data across age groups.

Taken together, the contributions in this Special Issue highlight the evolving scope of vaccine adjuvant science, from rational antigen–adjuvant co-design and mechanistic formulation studies to novel delivery platforms and lifespan-informed adjuvant strategy, in the shared pursuit of safer, more durable, and more broadly accessible vaccines.

## References

[1] Shi Z., Xi J., Cui M., Wan Z., Hou Y., Ma Z., Sun N., Zhu Y., Li M., Wang D. (2026). Rational Combination of Dominant and Subdominant Antigens with Nanoadjuvant Elicits Durable Immunity Against *Staphylococcus aureus*. Vaccines.

[2] Zdrehus R.-S., Mocan T., Sabau L.I., Matea C.T., Tăbăran F., Pop T., Delcea C., Mosteanu O., Mocan L. (2025). CEA-Functionalized Gold Nanoparticles as a Nanovaccine Platform: In Vitro Evaluation of Cytocompatibility, Cellular Uptake, and Antigen Processing. Vaccines.

[3] Ghattas M., Dwivedi G., Chevrier A., Scobey T., El-Mayta R., Mattocks M.D., Wang D., Lavertu M., Alameh M.-G. (2025). Comparative Analysis of Chitosan, Lipid Nanoparticles, and Alum Adjuvants in Recombinant SARS-CoV-2 Vaccine: An Evaluation of Their Immunogenicity and Serological Efficacy. Vaccines.

[4] Kanjo K., Lothe R., Nagar G., Rajurkar M., Rao H., Batwal S., Shaligram U., Varadarajan R. (2025). Destabilising Effect of Class B CpG Adjuvants on Different Proteins and Vaccine Candidates. Vaccines.

[5] D’onofrio V., Jacobs B., Alhatemi A., De Gussem S., Verstraete M., Porrez S., Willems A., De Boever F., Waerlop G., Leroux-Roels G. (2025). A novel carbohydrate fatty-acid monosulphate ester, squalane-in-water adjuvant is safe and enhances inactivated influenza vaccine immunogenicity in older adults. Vaccines.

[6] Arte T.M., Patil S.R., Adediran E., Singh R., Bagwe P., Gulani M.A., Pasupuleti D., Ferguson A., Zughaier S.M., D’souza M.J. (2025). Microneedle Delivery of Heterologous Microparticulate COVID-19 Vaccine Induces Cross Strain Specific Antibody Levels in Mice. Vaccines.

[7] Yao S., Zhai Z., Liao L., Zhong L., Shi C., Cheng Y.-X., Liu X. (2026). Intranasal Vaccine Adjuvants and Delivery Platforms: From Barrier Mechanisms to Clinical Translation. Vaccines.

[8] Singh S., Gautam S., Thakkar V., Kumar S., Joshi D. (2026). Viral Vaccine Adjuvant Strategies for Shaping Durable Immunity Across the Human Lifespan. Vaccines.

